# Editorial: Rising stars in cognition: 2023/4

**DOI:** 10.3389/fcogn.2025.1644789

**Published:** 2025-07-10

**Authors:** Melissa R. Beck, George R. Mangun

**Affiliations:** ^1^Department of Psychology, Louisiana State University, Baton Rouge, LA, United States; ^2^Department of Psychology and Neurology, University of California Davis, Davis, CA, United States

**Keywords:** memory, learning, forgetting, cortical oscillations, spatial processing, navigation, dendritic, neural synchrony oscillations

We are excited to introduce the *Rising Stars in Cognition 2023/2024* articles. The six articles in this Research Topic highlight research from early-career researchers that are using cutting edge techniques and studying topics critical to the development of our understanding of cognition. Across these papers we learn about (1) a model of dendritic processing of spatial sequences (2) cortical oscillations in spatial navigation (3) the limits of learning complex tonal structures (4) the neural process of forgetting in visual working memory (5) similarities and differences in brain activation as episodic memories are transferred from person to person and (6) neural synchrony between children and parents. Now, let's delve a bit deeper into each of these advancements in the science of cognition.

Our first rising star author is Dr. Johannes Leugering, a postdoctoral researcher at the University of California San Diego. Leugering et al. provides a model of how dendritic branches of neurons can represent sequential information that unfolds over variable time windows. This work highlights the important role of dendritic plateaus in neural processing of spatiotemporal information. Prior to this model, it was unclear how spatiotemporal properties could be represented in neural computation. Important features of the model that allow for flexibility are sensitivity to the order of locations visited during spatial navigation and flexibility across changes in speed. This allows the pattern of local dendritic plateaus to process the spatial order even over different time variations. Generally, this model provides an example of how single neurons can represent complex sequential information.

Expanding from the level of the dendrite to oscillations across areas of the brain, our second rising star author is Dr. Connor Thornberry, an Assistant Professor at the National College of Ireland. Thornberry and Commins looked at EEG oscillation patterns during spatial navigation. This has been done before intra-cranially, but not cortically. Delta and theta oscillations were higher for spatial navigation when learning to find a target compared to random non-goal directed exploration. Further, theta oscillations were more prominent in the beginning of the navigation process, while delta oscillations became more apparent later in navigation when focused search was needed. In addition, beta and gamma oscillations were higher in spatial navigation learning than in random exploration. As was seen with delta oscillations, beta oscillations were higher in the later, more focused stage of navigation. This occurred primarily in parieto-occipital locations and may be due to the involvement of memory in navigation. Generally, alpha oscillations did not appear to play a large role in the spatial navigation task.

Not only are spatial patterns and processing important to cognition, but Dr. Sauvé, a senior lecturer at the University of Lincoln, reveals the importance of patterns in tonal structures. Sauvé et al. examined the limits of statistical learning for auditory sequences. To test statistical learning of a complex structure (tonal hierarchy) for which participants had no prior knowledge, the authors used a scale that westerners are not exposed to (Bohlen-Pierce scale) but that was as complex as the western tonal scale. Generally, previous literature has shown quick learning (20 min) of simple tonal structures, however in the current study using complex structure, learning was not found after a 30-min exposure. This suggests that quick learning in previous studies may have been due to the simplistic structure. The authors also used qualitative measures to examine participants' reported strategies and awareness of the tonal properties. The themes detected were not supported by the quantitative data (goodness of fit ratings). This suggests a potential disconnect between explicit awareness and implicit processing.

Our rising star authors so far have focused on how the brain represents or learns information, but Dr. Moen, an Associate Professor at the University of Nebraska Kearney, asks how information is forgotten. Moen et al., used fMRI to examine neural activity during forgetting in visual working memory. Often information encoded in visual working memory can then be discarded as it is deemed no longer task relevant. This process may occur actively (requiring resources) or passively (removing resources). This study is innovative in examining forgetting in visual working memory while measuring brain activity as most prior work has looked at forgetting in long-term memory and/or has relied on behavioral evidence which can potentially be interpreted as supporting either passive or active forgetting. The results suggest that novel information can be discarded from visual working memory via a passive process where control resources are removed from maintaining the information, although an active process could not be ruled out.

While most of the work in cognition, and indeed the previous four rising star authors, focuses on processing in an individual's brain, Dr. Manuel Morante, a postdoctoral researcher at Aarhus University, tackles an important new frontier of understanding the coordination of brain activity between individuals. Morante et al. examine the encoding and reconstruction, through verbal reporting, of episodic memories across people using functional connectivity temporal analysis in fMRI data. The results reveal the importance of the default mode network and connections between the temporal lobe and the frontal lobe. The authors used the multiscale functional connectivity technique which is novel in its use of intrinsic oscillations in the fMRI data across timescales rather than static representations. This research furthers our understanding of how episodic memories are transferred across people and the similarities and differences in brain processing between the original encoding in one person and the reconstruction in another person.

The quest on our new frontier of understanding coordination of brain activity between individuals is continued with our final author, Dr. Yang Qu, an Assistant Professor at Northwestern University. In Qu et al., we find a review of the current literature examining an innovative and burgeoning area of research: neural synchrony between children and parents. The authors review research documenting the relationship between synchrony and wellbeing and other positive child outcomes. They note research showing that there are specific conditions that can predict increased synchrony and specific outcomes or consequences of synchrony that can be predicted. For example, synchrony is higher when there is a positive relationship between the parent and child, and synchrony is lower when there is a stressful family environment. Furthermore, if a child has more autonomy in a task, synchrony is higher. Synchrony can be recorded on a temporal scale using temporally sensitive measures like EEG or on a structural/functional scale using fMRI. This is an exciting area for development of new techniques for measuring synchrony and further understanding the predictors and outcomes of synchronicity.

In conclusion, our rising star authors present advances in our understanding of cognition on the level of the dendrite to the level of coordinating brain activity across individuals. We also gain knowledge on coding and detecting patterns in visual and auditory spaces and on the functions and sharing of working memory and episodic memory. Across these topics and techniques much is learned about cognition and foundations are set for new developments in the science of cognition.

